# Reduktion der Tinnituslautstärke: Pilotstudie zur Abschwächung von tonalem Tinnitus mit schwellennahem, individuell spektral optimiertem Rauschen

**DOI:** 10.1007/s00106-020-00963-5

**Published:** 2020-11-13

**Authors:** A. Schilling, P. Krauss, R. Hannemann, H. Schulze, K. Tziridis

**Affiliations:** 1 Experimentelle HNO-Heilkunde, HNO-Klinik, Kopf- und Halschirurgie, Universitätsklinikum Erlangen, Waldstraße 1, 91054 Erlangen, Deutschland; 2 WSAudiology, Sivantos GmbH, R&D AAA SA ERL, Erlangen, Deutschland

**Keywords:** Stochastische Resonanz, Reintonaudiometrie, Tinnitusfragebögen, Hörverlust, Individualtherapie, Stochastic resonance, Pure tone audiometry, Tinnitus questionnaires, Hearing loss, Individualized therapy

## Abstract

**Hintergrund:**

Tinnitus betrifft ca. 15 % der Bevölkerung, jedoch existiert noch immer kein echtes Heilverfahren. Ein von uns entwickeltes neuartiges Erklärungsmodell erlaubt nun die Erprobung einer gezielten, an den Ursachen der Tinnitusentstehung ansetzenden Behandlung. Diese basiert auf stochastischen Resonanzphänomenen an bestimmten synaptischen Verbindungen im Hörsystem, welche gezielt durch extern zugeführtes schwellennahes Rauschen induziert werden sollen.

**Fragestellung:**

Die vorliegende Pilotstudie soll zeigen, ob ein spektral individuell angepasstes Rauschen erfolgreich chronischen tonalen/schmalbandigen Tinnitus während der Stimulation abschwächen kann.

**Material und Methoden:**

Bei 22 volljährigen Tinnituspatienten (46.6±16.3 Jahre; 4 Frauen) wurden Hörverlust (HV) sowie Tinnitusfrequenzen (TF) und -lautstärken (TL) audiometrisch bestimmt. Darauf basierend wurden bis zu 8 verschiedene Rauschstimuli (RS) mit je 5 Lautstärken (−20 bis +20 dB SL) erzeugt. Diese wurden über audiologische Kopfhörer in einer Schallkammer für jeweils 40 s präsentiert. Nach jeder Präsentation wurde mithilfe einer 5‑stufigen Bewertungsskala (−2 bis +2) ermittelt, ob sich die TL verändert hat.

**Ergebnisse:**

Es fanden sich Patienten ohne Verbesserung der TL (*n* = 6) und solche mit Verbesserung (*n* = 16), wobei hier RS um die TF besonders effektiv waren. Die Gruppen zeigten post hoc deutliche Unterschiede in den Audiogrammen: Offenbar ist das hier getestete Verfahren insbesondere bei normalhörenden Tinnituspatienten und solchen mit geringgradigem HV effektiv.

**Schlussfolgerung:**

Die subjektiv wahrgenommene TL war bei 16 von 22 Probanden für die Dauer der Stimulation reduziert. Für den möglichen Erfolg einer zukünftigen Therapie scheint der HV relevant zu sein.

## Abstract (en)

**Background:**

Around 15% of the general population is affected by tinnitus, but no real cure exists despite intensive research. Based on our recent causal model for tinnitus development, we here test a new treatment aimed at counteracting the perception. This treatment is based on the stochastic resonance phenomenon at specific auditory system synapses that is induced by externally presented near-threshold noise.

**Objective:**

This pilot study will investigate whether individually spectrally adapted noise can successfully reduce chronic tonal/narrow-band tinnitus during stimulation.

**Materials and methods:**

Hearing loss (HL) as well as tinnitus pitch (TP) and loudness (TL) were audiometrically measured in 22 adults (46.6±16.3 years; 4 women) with tinnitus. Based on these measurements, up to eight different noise stimuli with five intensities (−20 to +20 dB SL) were generated. These were presented for 40 s each via audiologic headphones in a soundproof chamber. After each presentation, the change in TL was rated on a five-level scale (−2 to +2).

**Results:**

We found patients (*n* = 6) without any improvement in their TL perception as well as patients with improvement (*n* = 16), where stimulation around the TP was most effective. The groups differed in post-hoc analysis of their audiograms: the effectiveness of our new therapeutic strategy obviously depends on the individual HL, and was most effective in normal-hearing tinnitus patients and those with mild HL.

**Conclusion:**

Subjective TL could be reduced in 16 out of 22 patients during stimulation. For a possible success of a future therapy, the HL seems to be of relevance.

Nach gängigen Modellen [13] resultiert Tinnitus zumeist aus einem Hörverlust (HV), wobei die tinnitusbedingte Belastung mitunter schwerwiegender sein kann als der eigentliche HV. Die Behandlung beschränkt sich oft auf Bewältigungsstrategien, da Mechanismen der Tinnitusentstehung noch immer umstritten sind. In dieser Pilotstudie testen wir an Patienten einen neuartigen Ansatz einer möglichen zukünftigen Therapie, den wir auf der Grundlage unseres Modells der Tinnitusentstehung durch stochastische Resonanz (SR) [14] entwickelt haben und der an den von uns postulierten Ursachen derselben ansetzt.

Neben einigen Erfolgen bei der Linderung von Tinnitus in jüngerer Zeit [1, 19] ist ein wesentlicher Grund dafür, dass es bis heute kein an den Ursachen der Entstehung ansetzendes Heilverfahren zur Behandlung von chronischem Tinnitus gibt, die Uneinigkeit der Forschung über die zu seiner Entstehung führenden neurophysiologischen Mechanismen. Gängige Modelle zur Entstehung der verschiedenen, teilweise sehr heterogenen Tinnitusperzepte [5] gehen zwar nahezu alle davon aus, dass Tinnitus infolge eines (sehr geringen) Hörschadens entsteht, konnten bislang aber lediglich Teilaspekte des Phänomens erklären [2, 6, 9, 16, 18, 20, 21]. Unlängst haben wir ein neuartiges, mechanistisches Modell der Tinnitusentstehung, basierend auf tierexperimentellen Daten und der Modellierung neuronaler Netze, entwickelt [10, 12, 14, 15]. Es macht überprüfbare Vorhersagen und impliziert eine völlig neuartige Behandlungsstrategie gegen tonalen bzw. schmalbandigen Tinnitus. Ziel der vorliegenden Pilotstudie ist ein Proof of Concept dieses neuartigen Ansatzes.

Im Modell wird angenommen, dass die Tinnitusentstehung eine Begleiterscheinung des Versuchs des Gehirns ist, einen entstandenen Hörverlust auszugleichen. Dabei bedient sich das Hörsystem laut Modell des Phänomens der SR: Hierbei kann ein primär unterschwelliges Signal durch Beimischen von Rauschen geeigneter Intensität über die Sensor-Detektionsschwelle gehoben und so messbar gemacht werden [3, 8]. Wir konnten zeigen, dass sich die optimale Intensität des beizumischenden Rauschens mittels Autokorrelation des Sensoroutputs bestimmen lässt [12]. Das Modell [14] nimmt daher an, dass das Hörsystem durch Erhöhung von internem, neuronalem Rauschen SR auslöst und so das Hörvermögen nach Hörschaden sekundär wieder verbessert. Das hierfür nötige interne Rauschen wäre dann als Tinnitus wahrnehmbar.

An audiometrischen Daten von fast 40.000 Patienten konnten wir zeigen, dass Tinnituspatienten im sprachrelevanten Frequenzbereich bis 3 kHz tatsächlich signifikant bessere Hörschwellen aufwiesen als Patienten ohne Tinnitus [10]. Da das Modell somit eine konkrete Ursache für die als Tinnitus wahrgenommene neuronale Aktivität postuliert – das intern generierte neuronale Rauschen – haben wir eine neuartige Behandlungsstrategie gegen Tinnitus entworfen. Der Grundgedanke besteht darin, das laut Modell vom Hörsystem zur Verbesserung des Hörvermögens generierte und als Tinnitus wahrgenommene interne neuronale Rauschen durch extern beigemischtes akustisches Rauschen zu ersetzen. Dass es tatsächlich möglich ist, die Hörschwellen normalhörender Probanden durch extern beigemischtes Rauschen zu verbessern, konnte bereits gezeigt werden [22].

Das Neuartige an unserer Idee ist, dass – im Gegensatz z. B. zu herkömmlichen Rauschgeneratoren zur Tinnitusmaskierung – das SR-auslösende beigemischte Rauschen selbst nur knapp über- oder sogar unterschwellig sein muss, um das interne neuronale Rauschen überflüssig zu machen. Dieses externe Rauschen, so unser Ansatz, könnte dann anstelle des internen Rauschens die Hörschwellen verbessern, sodass für das Hörsystem die Notwendigkeit entfiele, internes Rauschen zu nutzen, wodurch in der Folge auch das darauf beruhende Tinnitusperzept verschwinden oder zumindest abgeschwächt werden sollte.

## Methoden

### Probanden

Es wurden 22 erwachsene Tinnitusprobanden (4 Frauen) mit Einverständnis (Ethikkommission des UK Erlangen: AZ 159_18) untersucht. Ihr mittleres Alter (±Standardabweichung) war 46,6 (±16,3) Jahre, der Median (unteres, oberes Quartil) der bestimmten Tinnitusfrequenz (TF) betrug 6 (4, 8) kHz. Alle Probanden hatten einen Reinton- bzw. schmalbandigen Tinnitus. Zwölf von 22 Probanden (vgl. auch Tab. 1) zeigten eine binaurale, meist nahezu symmetrische, 4 Probanden eine monaurale Mittel- bis Hochtonschwerhörigkeit (Hörverlust ≥20 dB), ein Proband war einseitig taub und monaural schwerhörig und 5 Probanden waren klinisch normalhörend. Alle Tinnitusschweregrade (SG; Mini-Tinnitus-Fragebogen, Mini TF12: SG I: 8; SG II: 4; SG III: 5; SG IV: 4; ein Fragebogen wurde nicht ausgefüllt; vgl. Tab. 1) waren vertreten.

**Table 1:** 

Prob. Nr.	Seite (Ohr)	Frequenz (kHz)												MW HV (dB)	TF (kHz)	TL (dB)	TL (dB SL)	SG	Alter
		0,125	0,25	0,5	0,75	1	1,5	2	3	4	6	8	10						
*1*	*R*	5	6	5	4	3	9	8	2	3	8	10	–	5,7	2	12	4	4	22
	*L*	8	7	4	4	5	5	4	1	8	10	22	–	7,1	2	6	2		
*2*	*R*	9	10	9	7	10	22	38	56	71	71	75	–	34,4	8	74	3	2	79
	*L*	12	13	14	11	10	20	29	53	58	65	70	–	32,3	–	–	–		
*3*	*R*	6	3	3	5	5	4	4	3	3	7	7	–	4,5	3	14	11	3	22
	*L*	13	9	5	5	6	4	4	5	5	5	10	–	6,5	2	13	9		
*4*	*R*	10	5	5	3	3	4	4	4	6	6	9	–	5,4	6	11	5	1	26
	*L*	5	5	3	3	3	1	2	3	2	7	8	–	3,8	4	10	8		
*5*	*R*	4	0	1	2	3	2	2	14	19	10	18	–	6,8	–	–	–	3	54
	*L*	9	3	3	4	5	7	9	39	32	58	53	62	23,7	10	76	14		
*6*	*R*	11	8	8	7	7	7	7	8	15	10	10	–	8,9	–	–	–	1	27
	*L*	11	8	5	5	4	5	5	8	12	38	37	46	15,3	10	46	0		
*7*	*R*	8	7	5	6	5	8	8	8	18	19	41	59	16,0	6	21	2	4	38
	*L*	9	9	8	10	8	13	18	37	44	48	28	21	21,1	–	–	–		
*8*	*R*	8	3	3	5	6	13	10	27	33	59	57	72	24,7	6	60	1	–	53
	*L*	9	8	11	14	7	7	13	29	43	61	70	78	29,2	6	64	3		
*9*	*R*	11	10	10	13	20	25	29	46	48	44	48	–	27,6	8	59	11	3	50
	*L*	12	10	11	14	22	34	39	49	46	40	40	–	28,8	8	46	6		
*10*	*R*	11	7	3	3	6	11	15	14	2	79	81	–	21,1	8	82	1	2	54
	*L*	10	5	5	5	7	15	22	29	21	38	50	–	18,8	8	50	0		
*11*	*R*	12	9	8	8	7	7	7	9	18	35	32	–	13,8	0,125	15	3	4	49
	*L*	10	8	4	4	3	7	7	12	20	44	35	–	14,0	8	65	30		
*12*	*R*	3	2	1	3	4	3	0	15	24	28	17	32	11,0	–	–	–	2	40
	*L*	10	4	7	10	10	11	19	47	61	62	62	66	30,8	6	63	1		
*13*	*R*	9	9	10	18	25	44	58	59	54	64	62	–	37,5	6	66	2	2	53
	*L*	–	–	–	–	–	–	–	–	–	–	–	–	–	–	–	–		
*14*	*R*	8	7	7	10	13	18	9	8	30	11	40	–	14,6	–	–	–	3	59
	*L*	11	12	17	19	19	25	18	19	29	58	72	–	27,2	0,125	20	9		
*15*	*R*	15	16	11	17	15	10	17	19	24	17	12	–	15,7	4	25	1	1	47
	*L*	21	20	12	17	19	14	18	21	21	27	13	–	18,5	4	22	1		
*16*	*R*	–	11	13	10	10	10	10	9	11	14	10	–	10,8	13,8	10	–	1	46
	*L*	–	17	13	12	11	11	14	14	14	20	14	–	14,0	13,8	12	–		
*17*	*R*	15	8	8	8	9	8	10	10	11	12	10	–	9,9	6	13	1	3	40
	*L*	9	7	7	6	9	9	18	9	20	18	9	–	11,0	4	26	6		
*18*	*R*	18	22	21	24	30	27	18	29	41	68	96	–	35,8	6	74	6	1	63
	*L*	15	16	12	13	18	25	34	64	67	86	90	–	40,0	6	87	1		
*19*	*R*	6	6	7	7	8	9	8	7	7	7	12	–	7,6	8	9	−3	1	24
	*L*	8	5	5	6	6	6	6	6	6	10	10	–	6,7	8	17	7		
*20*	*R*	13	12	10	11	15	20	30	41	58	63	75	73	35,1	–	–	–	4	72
	*L*	9	8	10	10	16	16	31	52	50	71	82	76	35,9	10	53	−23		
*21*	*R*	13	13	10	9	11	19	21	20	20	24	27	–	17,0	6	28	4	1	36
	*L*	19	19	15	17	15	12	16	20	57	78	60	–	29,8	–	–	–		
*22*	*R*	19	11	12	9	9	9	7	30	28	31	37	41	18,4	6	32	1	1	73
	*L*	13	12	13	9	9	9	27	31	30	31	35	38	19,9	6	34	3		

### Audiometrie und Rauschanpassung

Die Probanden wurden in der Audiologie der HNO-Klinik, Kopf- und Halschirurgie des UK Erlangen binaural audiologisch untersucht und sowohl die Reinton-Hörschwellen sowie die TF zwischen 0,125 und 10 kHz nach ISO 8253‑1 bestimmt (Ausnahme Proband 16, TF von externem Audiologen bestimmt). Basierend auf diesen Daten wurden zwischen 6 und 8 (abhängig von ihrer TF) Rauschstimuli (RS) mit jeweils 5 Lautstärken (−20 bis +20 dB SL, 10-dB-Schritte) mittels eines selbst geschriebenen Python-Programms (Python 3.6, Numpy Bibliothek; Anaconda Distribution, Anaconda, Berlin, Deutschland) erzeugt. Zusätzlich wurde ein Durchlauf ohne Stimulation (Stille) erzeugt. Die verschiedenen RS waren: weißes Rauschen (WR), ein tiefpassgefiltertes (TPR) sowie ein hochpassgefiltertes Rauschen (HPR), jeweils mit der Grenzfrequenz bei der TF. Bis zu 5 verschiedene Schmalband gefilterte Rauschen (SBR) wurden verwendet mit jeweils einer Breite von ±½ Oktave. Die Mittenfrequenzen dieser SBR reichten von einer Oktave unterhalb der TF bis eine Oktave oberhalb der TF mit einer Schrittweite von ½ Oktave. In seltenen Ausnahmen wurden RS mit Mittenfrequenzen außerhalb dieser Spezifikationen verwendet (z. B. TF >8 kHz). Die Lautstärke der RS wurde entsprechend der Audiogramme der Ohren der Probanden gewählt, wobei bei unterschiedlichen Audiogrammen das Tinnitus-Ohr gewählt wurde; bei beidseitigem Tinnitus wurde das Audiogramm des besseren Ohrs verwendet. Für die Lautstärke des WR wurde der Audiogramm-Mittelwert aller Frequenzen verwendet, bei den HPR und TPR die Audiogramm-Mittelwerte der beinhalteten Frequenzen und bei den SBR wurde die Hörschwelle der Mittenfrequenz als 0 dB SL Referenz gewählt.

### Experimentelle Durchführung

Die Stimuli wurden den Probanden an einem gesonderten Messtag über audiologische Kopfhörer in einer Schallkammer für jeweils 40 s einmal präsentiert, nach jeder Präsentation wurden sie vom Versuchsleiter gefragt, ob sich die TL verändert habe. Zur Eingewöhnung wurden die WR-Stimuli immer zuerst in absteigender Lautstärke präsentiert, mit Stille am Ende dieser Reihe. Danach wurden die individuell gefilterten RS in aufsteigender Lautstärke und Frequenz präsentiert. Die Probanden waren instruiert, nur auf ihren Tinnitus zu achten und konnten mit einer von 5 möglichen gewerteten Antworten antworten: „Tinnitus war deutlich lauter“ (−2), „Tinnitus war etwas lauter“ (−1), „TL hat sich nicht verändert“ (0), „Tinnitus war etwas leiser“ (+1) und „Tinnitus war deutlich leiser“ (+2). Zusätzlich konnten sie auch weitere Angaben machen (z. B. empfundene Maskierungseffekte) und wurden vor dem Versuch auch entsprechend instruiert. Die Messung aller Stimuli dauerte zwischen 45 und 60 min, Pausen waren möglich, wurden aber selten genutzt. Jeder Proband erhielt € 50,- Aufwands- sowie eine Fahrtkostenerstattung nach Abschluss der Experimente.

### Statistische Auswertung

Die Daten wurden mittels Statistica 8 (Fa. StatSoft, Hamburg) ausgewertet. Zunächst wurden die Daten nach den individuellen Berichten der Probanden klassifiziert. Für alle Analysen der Hörschwellen wurden nur die von Tinnitus betroffenen Seiten (Ohren) ausgewertet. Es fanden sich 2 Gruppen von Probanden: Zum einen die „Nichtresponder“ (NR). Diese Probanden zeigten bei keiner der Stimulationen eine subjektive Verbesserung ihres Tinnitusperzepts (*n* = 6; 10 Tinnitus-Ohren, 27,3 % [29,4 %]). Bei ihnen kam es also bestenfalls zu Maskierungen. Zum anderen die „Responder“ (R; *n* = 16; 24 Tinnitus-Ohren, 72,7 % [70,6 %]), also die Probanden, die mindestens bei einem RS eine Antwort mit dem Wert +1 gaben, also eine subjektive Verbesserung ihres Tinnitusperzepts ohne Maskierung. Basierend auf dieser Klassifikation wurden die Hörschwellen der Tinnitus-Ohren (22 Probanden: 43 Ohren gesamt, 34 Tinnitus-Ohren, 79,1 %, ausgewertet) sowie die Antworten der Probanden auf die verschiedenen Rauschreize mittels ANOVA bzw. Kruskal-Wallis(KW)-ANOVA statistisch ausgewertet.

## Ergebnisse

### Hörverlust

**Figure 1:**
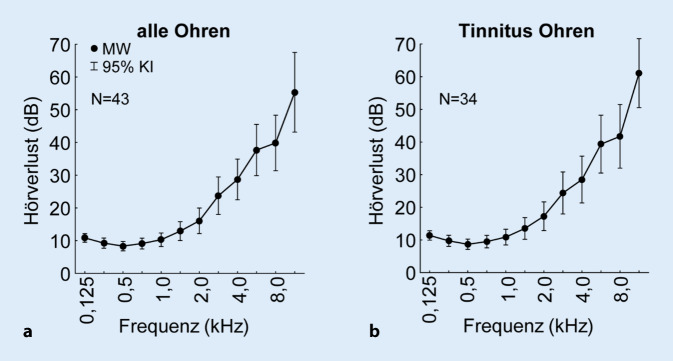

In Tab. 1 ist der audiologisch gemessene Hörverlust (HV) in dB für jeden Probanden angegeben. Der Mittelwert des HV (MW HV) ist über alle gemessenen Frequenzen für jedes Ohr berechnet und die Grundlage für die Lautstärke des WR. In den letzten Spalten ist die TF in kHz sowie die TL in dB und dB SL angegeben. Als zusätzliche Information sind in den letzten beiden Spalten der SG sowie das Alter der Probanden angegeben. Die mittleren HV (±95%-Konfidenzintervall, 95%-KI) aller 22 Probanden (43 Ohren) sind in Abb. 1a gezeigt; Abb. 1b zeigt den HV der 34 Tinnitus-Ohren. Nur diese Ohren wurden für die weiteren Analysen verwendet.

### Tinnitus und Klassifizierung der Probanden

Die TF der 22 Probanden lag im Median (±Interquartilsabstand) bei 6 (4, 8) kHz. Die TF zeigten keine signifikanten Unterschiede in Abhängigkeit vom SG (KW-ANOVA: H(3, 29) = 2,26; *p* = 0,52). Die TL (dB SL) dagegen zeigte eine signifikante Abhängigkeit von diesem (KW-ANOVA: H(3, 29) = 9,32; *p* = 0,025) mit der größten TL bei SG III im Vergleich zu SG II (multiple Vergleiche der mittleren Ränge, *p* = 0,045), wobei die TL bei allen anderen SG nicht signifikant unterschiedlich voneinander waren. TF und TL (dB SL) zeigten keinen Zusammenhang (KW-ANOVA: H(12, 33) = 4,95; *p* = 0,55).

Die Tab. 2 zeigt Übersichtsdaten der Probandenantworten: individuelle Antwort-Summe über alle 5 Lautstärken (Minimum: −10; Maximum: +10), sowie in Klammern Minimum und Maximum der gegebenen Antworten (−2 bis +2) bei den verschiedenen RS (bei den SBR entspricht die Zahl dahinter dem Abstand der Mittenfrequenz von der TF in Oktaven). Eine eventuelle Maskierung und die entsprechende Klassifizierung in NR und R (vgl. Methoden) folgt. Bei Stille war nur eine Antwort möglich, sodass hier die Summe von −2 bis +2 geht und es kein Minimum und Maximum gibt.

**Table 2:** 

Rauschtyp	Probandennummer																					
	1	2	3	4	5	6	7	8	9	10	11	12	13	14	15	16	17	18	19	20	21	22
*Stille*	0	0	0	0	0	0	0	0	0	0	0	0	0	0	0	0	0	0	0	0	0	0
*WR*	0 (0,0)	0	4 (0,2)	4 (0,2)	1 (0,1)	1 (0,1)	0 (0,0)	0 (0,0)	0 (0,0)	1 (0,1)	−1 (−1,0)	0 (0,0)	0 (0,0)	2 (0,1)	2 (0,2)	0 (0,0)	−2 (−1,0)	0 (0,0)	0 (0,0)	0 (0,0)	0 (0,0)	0 (0,0)
*HPR*	−1 (−1,0)	0 (0,0)	−1 (−1,0)	2 (0,1)	4 (0,2)	3 (0,1)	1 (0,1)	0 (0,0)	0 (0,0)	0 (0,0)	−1 (−1,0)	0 (0,0)	0 (0,0)	2 (0,1)	5 (0,2)	–	2 (0,2)	0 (0,0)	0 (0,0)	–	1 (0,1)	2 (0,1)
*TPR*	–	0 (0,0)	4 (0,1)	–	0 (0,0)	0 (0,0)	1 (0,1)	0 (0,0)	0 (0,0)	−1 (−1,0)	−2 (−1,0)	5 (0,1)	–	–	–	0 (0,0)	–	–	3 (0,1)	0 (0,0)	–	–
*SBR‑2*	–	–	–	–	–	–	–	–	–	–	–	–	–	–	–	0 (0,0)	–	–	–	0 (0,0)	–	–
*SBR‑1,5*	–	–	–	–	–	–	–	–	–	–	–	–	–	–	–	0 (0,0)	–	–	–	0 (0,0)	–	–
*SBR‑1*	0 (0,0)	0 (0,0)	2 (0,1)	0 (0,0)	1 (0,1)	2 (0,1)	−1 (−1,0)	0 (0,0)	0 (0,0)	2 (0,1)	−1 (−1,0)	1 (0,1)	0 (0,0)	–	4 (0,2)	0 (0,0)	1 (0,1)	0 (0,0)	−3 (−1,0)	0 (0,0)	1 (0,1)	2 (0,1)
*SBR‑0,5*	0 (−1,1)	0 (0,0)	3 (0,1)	1 (−1,1)	6 (0,2)	2 (0,1)	1 (0,1)	0 (0,0)	0 (0,0)	4 (0,1)	0 (0,0)	2 (0,1)	0 (0,0)	–	0 (0,0)	0 (0,0)	2 (0,1)	0 (0,0)	0 (0,0)	0 (0,0)	4 (0,2)	4 (0,2)
*SBR 0*	0 (−1,1)	0 (0,0)	4 (0,2)	0 (0,0)	4 (0,2)	2 (0,1)	5 (0,2)	0 (0,0)	0 (0,0)	5 (0,2)	0 (−1,1)	1 (0,1)	3 (0,2)	4 (0,2)	0 (0,0)	–	3 (0,1)	0 (0,0)	−1 (−1,0)	0 (0,0)	2 (0,2)	4 (0,2)
*SBR +0,5*	0 (0,0)	0 (0,0)	0 (0,0)	1 (0,1)	–	–	–	0 (0,0)	0 (0,0)	0 (0,0)	0 (−1,1)	2 (0,1)	2 (0,2)	2 (0,2)	6 (1,2)	–	3 (0,2)	0 (0,0)	0 (0,0)	–	0 (0,0)	1 (0,1)
*SBR+1*	−1 (−1,0)	–	–	4 (0,2)	–	–	–	–	–	–	–	1 (0,1)	0 (0,0)	1 (0,1)	4 (0,2)	–	4 (0,2)	0 (0,0)	–	–	0 (0,0)	4 (0,1)
*SBR +1,5*	–	–	–	–	–	–	–	–	–	–	–	–	–	1 (0,1)	–	–	–	–	–	–	–	–
*Maskiert*	j	j	j	j	j	*n*	j	j	j	*n*	j	*n*	*n*	*n*	*n*	j	*n*	j	j	j	j	*n*
*Klasse*	R	NR	R	R	R	R	R	NR	NR	R	R	R	R	R	R	NR	R	NR	R	NR	R	R

### Klassifizierungsabhängige Analysen der Hörschwellen

**Figure 2:**
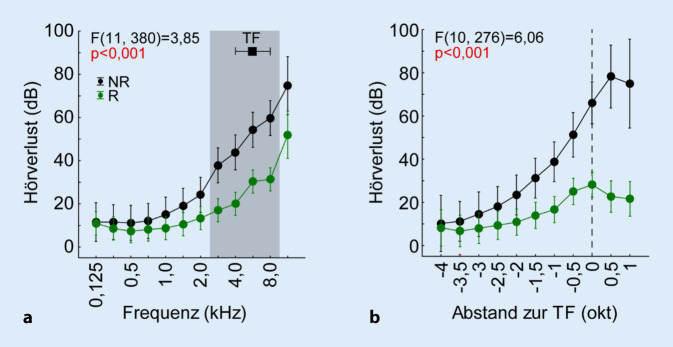

Basierend auf der Klassifizierung aus den Antworten der Probanden wurde der Hörverlust der Tinnitus-Ohren analysiert. Zunächst wurde eine 2‑faktorielle ANOVA (Faktoren *Frequenz* und *Gruppe*) der auf die Frequenzen alignierten individuellen HV-Daten durchgeführt. Die Probanden zeigten im Mittel eine Hochtonschwerhörigkeit (*Frequenz*: F(11, 380) = 34,98; *p* < 0,001). Der Faktor *Gruppe* zeigte ebenfalls einen Einfluss auf den Hörverlust (F(1, 380) = 69,98; *p* < 0,001) mit den Mittelwerten (±95%-KI) der Gruppe NR 31,3 (±4,4) dB und R 18,2 (±1,9) dB. Die signifikante Interaktion (F(11, 380) = 3,85; *p* = 0,001) der beiden Faktoren (Abb. 2a) zeigt die Staffelung des HV der beiden Gruppen frequenzabhängig auf. Erst ab den Frequenzen oberhalb von 3 kHz zeigen sich die HV in den Tukey-Post-hoc-Tests (*p* < 0,05) zwischen NR- und R‑Probanden signifikant.

In einer weiteren 2‑faktoriellen ANOVA (Faktoren: *Abstand zur TF* sowie *Gruppe*) wurden die HV-Daten auf die individuelle TF aligniert und der Abstand der Frequenzen in Halboktaven dazu berechnet. Es zeigte sich auch hier eine Abhängigkeit des HV vom Abstand zur TF (F(10, 276) = 22,3; *p* < 0,001), wobei sich ein Plateau von −1 okt TF bis +1 okt TF erstreckt, das einen signifikant größeren HV im Bereich der TF im Vergleich zu den tieferen Frequenzen zeigt (Tukey-Post-hoc-Tests, *p* < 0,05). Innerhalb des Plateaus zeigen sich keine signifikanten Unterschiede im HV. Auch hier unterscheiden sich die mittleren HV der 2 Gruppen signifikant (F(2, 276) = 115,2; *p* < 0,001) mit NR 38,0 (±5,5) dB und R 15,6 (±2,4) dB. Auch die Interaktion der beiden Faktoren (Abb. 2b) zeigt einen signifikanten frequenzabhängigen Einfluss der Gruppen auf den HV (F(10, 276) = 6,06; *p* < 0,001). Die beiden Gruppen unterscheiden sich dabei in der Kurvenform deutlich (Tukey-Post-hoc-Tests), die NR-Probanden zeigen ab +0,5 okt TF ein Plateau mit größtem HV und die R‑Probanden zeigen eine HV-Spitze zwischen −0,5 okt TF und der TF. Mit anderen Worten, die NR-Probanden haben einen großen HV, der vor allem oberhalb der TF zu finden ist, und die R‑Probanden zeigen einen moderaten HV, der knapp unterhalb und bei der TF am größten ist.

### Subjektive Veränderung der TL bei Respondern

**Figure 3:**
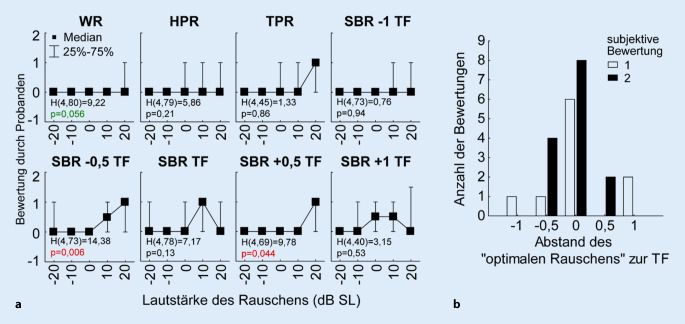

Die Responder berichteten Verbesserungen der TL durch bestimmte RS. Dies ist in Abb. 3 zusammengefasst. Die Analyse der Antworten innerhalb der einzelnen RS wurde mit KW-ANOVA mit dem Faktor *Rauschlautstärke* durchgeführt. Die Antworten aller 16 R-Probanden (Abb. 3a) zeigte in 2 der 8 RS eine signifikante Abhängigkeit von der Rauschlautstärke und in einem der 8 RS einen Trend dazu. Es fällt auf, dass die Mediane der Antworten erst ab +10 dB SL zu steigen scheinen (also eine subjektive Verringerung der TL anzeigen). Die Post-hoc-Statistik kann aber aufgrund der geringen Anzahl der Messungen (maximal 16 Datenpunkte pro Lautstärke) und der relativ hohen Streuung nur einen einzigen Trend (Median Tests für multiple Vergleiche) aufzeigen: SBR −0,5 okt TF: 0 dB vs. 20 dB; *p* = 0,066. Wie schon beim HV wurden die Antworten der Probanden auf ihre TF aligniert: der Rauschreiz mit der niedrigsten Cut-off- oder Mittenfrequenz und größten subjektiven Antwort wurde in Relation zur TF gesetzt. Dieses „optimale Rauschen“ lag zwischen −1 okt und +1 okt zur TF (Abb. 3b), wobei 14 der 24 möglichen positiven Antworten (58,3 %) direkt bei der TF lagen und insgesamt ebenfalls 14 der 24 Antworten mit einer Wertigkeit von +2 angegeben wurden.

## Diskussion

In dieser Pilotstudie konnte der Proof of Concept erbracht werden, dass spektral an den HV von Tinnituspatienten angepasstes, schwellennahes Rauschen in der Lage ist, ein Tinnitusperzept mindestens teilweise abzuschwächen, ohne es zu maskieren. Diese Stimulation scheint einen signifikanten Effekt zu haben, im Gegensatz zu Studien mit amplitudenmodulierten Reintönen [17]. Im Gegensatz zur herkömmlichen Maskierung, die durch ein lautes unspezifisches Rauschen den Tinnitus übertönen soll [4], zielt unser therapeutischer Ansatz darauf ab, die Entstehungsursache von Tinnitus im Gehirn, die laut unserem Modell primär der Verbesserung des Hörens (genauer, der Optimierung der Informationsübertragung zwischen Ohr und Gehirn) dient [14], überflüssig zu machen.

Einige Probanden der Responder-Gruppe 8/16 (50 %) berichteten bei bestimmten RS auch von Maskierungseffekten (R_mask_), meist beim WR (26 %), deutlich seltener bei anderen RS (zwischen 4 und 10 % je Stimulus). Von diesen Maskierungen traten 83,6 % bei Stimuli mit +10 oder +20 dB SL, also bei vergleichsweise hohen Lautstärken, auf. Die R_mask_-Probanden (vgl. Tab. 1) hatten einen signifikant geringeren mittleren HV (7,3 dB; t‑Test, *p* < 0,001) als die Probanden, die niemals über einen Maskierungseffekt berichteten. Wir schließen daraus, dass die sehr guten Hörschwellen der überwiegend normalhörenden Probanden dieser Subgruppe (R_mask_) dazu führten, dass bereits Stimuli mit +10 oder +20 dB SL eine deutliche Maskierung des Tinnitus bewirkten, dies aber nicht frequenzspezifisch, sondern vor allem bei WR-Stimulation auftrat. Alle Responder zeigten demgegenüber ihre besten Ergebnisse bei RS, welche spektral im Bereich der TF lagen, was den Vorhersagen aus unserem Modell entspricht [13]. Die NR-Probanden zeigten in der Audiometrie dagegen den größten HV. Offenbar stieß hier das von uns entwickelte akustische Stimulationsverfahren schlicht an seine Grenzen.

Dies zeigt auch eine der Limitationen dieser Pilotstudie. Wir untersuchten ein kleines Kollektiv mit breiter Altersverteilung und sehr unterschiedlichen Hörverlusten, Tinnitusfrequenzen und Belastungsgraden. Das Rauschen wurde in relativ groben 10-dB-Schritten und relativ breiten Frequenzspektren einmalig dargeboten. Allgemeine Aussagen zu allen Tinnitustypen lassen sich mit einer solchen Pilotstudie also nicht treffen, allerdings haben fast 73 % der Probanden mit Reinton‑/Schmalband-Tinnitus von einem positiven Effekt der Stimulation berichtet.

Unser Ansatz unterscheidet sich von herkömmlicher Maskierung und auch von sog. Residual-Inhibition-Ansätzen [7] vor allem durch die Lautstärke des dargebotenen RS (vgl. auch [11]). Das spektral individuell angepasste Rauschen liegt im Bereich der Hörschwelle oder wenige dB darüber. Die Probanden nehmen auch nur während der Stimulation eine Unterdrückung ihres Tinnitus wahr. Dies entspricht ebenfalls genau den Vorhersagen des Modells und ermöglicht es uns als nächsten Schritt, Lautstärke und Spektralzusammensetzung der RS noch genauer anzupassen, um dann perspektivisch Geräte mit dieser Technologie auszustatten.

## Fazit für die Praxis

Insgesamt 16/22 Probanden profitierten von der Stimulation, d. h. zeigten bei optimaler spektraler Zusammensetzung und Lautstärke des Rauschreizes eine Verringerung der Tinnituslautstärke, die nicht auf simpler Maskierung beruhte.

Wir schließen aus diesen Befunden, dass für einige Tinnituspatienten mit ausreichendem Hörvermögen ein kontinuierlich dargebotener Rauschstimulus so optimiert werden kann, dass das Tinnitusperzept im Idealfall ganz unterdrückt wird.

## References

[1] Adamchic I, Toth T, Hauptmann C (2017). Acute effects and after-effects of acoustic coordinated reset neuromodulation in patients with chronic subjective tinnitus. Neuroimage Clin..

[2] Ahlf S, Tziridis K, Korn S (2012). Predisposition for and prevention of subjective tinnitus development. Plos One.

[3] Benzi R, Sutera A, Vulpiani A (1981). The mechanism of stochastic resonance. J Phys A: Math Gen.

[4] Cai Y, Zhou Q, Yang H (2017). Logistic regression analysis of factors influencing the effectiveness of intensive sound masking therapy in patients with tinnitus. BMJ Open.

[5] Cederroth CR, Gallus S, Hall DA (2019). Towards an understanding of tinnitus heterogeneity. Front Aging Neurosci.

[6] Eggermont JJ, Roberts LE (2004). The neuroscience of tinnitus. Trends Neurosci.

[7] Fournier P, Cuvillier A-F, Gallego S (2018). A new method for assessing masking and residual inhibition of tinnitus. Trends Hear.

[8] Gammaitoni L, Hänggi P, Jung P (1998). Stochastic resonance. Rev Mod Phys.

[9] Gerken GM (1996). Central tinnitus and lateral inhibition: an auditory brainstem model. Hear Res.

[10] Gollnast D, Tziridis K, Krauss P (2017). Analysis of audiometric differences of patients with and without Tinnitus in a large clinical database. Front Neurol.

[11] Henry JA, Jastreboff MM, Jastreboff PJ (2002). Assessment of patients for treatment with tinnitus retraining therapy. J Am Acad Audiol.

[12] Krauss P, Metzner C, Schilling A (2017). Adaptive stochastic resonance for unknown and variable input signals. Sci Rep.

[13] Krauss P, Schilling A, Tziridis K (2019). Modelle der Tinnitusentstehung. HNO.

[14] Krauss P, Tziridis K, Metzner C (2016). Stochastic resonance controlled upregulation of internal noise after hearing loss as a putative cause of tinnitus-related neuronal hyperactivity. Front Neurosci.

[15] Krauss P, Tziridis K, Schilling A (2018). Cross-modal stochastic resonance as a universal principle to enhance sensory processing. Front Neurosci.

[16] Leaver AM, Turesky TK, Seydell-Greenwald A (2016). Intrinsic network activity in tinnitus investigated using functional MRI. Hum Brain Mapp.

[17] Neff P, Zielonka L, Meyer M (2019). Comparison of amplitude modulated sounds and pure tones at the tinnitus frequency: residual tinnitus suppression and stimulus evaluation. Trends Hear.

[18] Schaette R, Mcalpine D (2011). Tinnitus with a normal audiogram: physiological evidence for hidden hearing loss and computational model. J Neurosci.

[19] Stein A, Wunderlich R, Lau P (2016). Clinical trial on tonal tinnitus with tailor-made notched music training. BMC Neurol.

[20] Tziridis K, Ahlf S, Jeschke M (2015). Noise trauma induced neural plasticity throughout the auditory system of Mongolian gerbils: differences between Tinnitus developing and non-developing animals. Front Neurol.

[21] Vanneste S, De Ridder D (2016). Deafferentation-based pathophysiological differences in phantom sound: tinnitus with and without hearing loss. Neuroimage.

[22] Zeng F-G, Fu Q-J, Morse R (2000). Human hearing enhanced by noise1. Brain Res Brain Res Protoc.

